# Arterial-optimized 4D-flow MRI for quantifying flow and pulsatility in venous sinuses and large cerebral veins

**DOI:** 10.1038/s41598-025-28405-8

**Published:** 2025-11-20

**Authors:** Arvid Westlund, Anders Wåhlin, Jan Malm, Anders Eklund

**Affiliations:** 1 Clinical Sciences, Neurosciences, Umeå University, Umeå, 90187 Sweden; 2 Department of Diagnostics and Intervention, Biomedical Engineering and Radiation Physics, Umeå University, Umeå, 90187 Sweden; 3 Umeå Center for Functional Brain Imaging (UFBI), Umeå University, Umeå, 90187 Sweden; 4 Department of Applied Physics and Electronics, Umeå University, Umeå, 90187 Sweden

**Keywords:** 4D flow MRI, Cerebral sinus, Hemodynamics, Cerebral vein, Velocity encoding, Neurology, Biomedical engineering

## Abstract

A single, arterial-optimized 4D-flow MRI acquisition may enable fast assessment of both cerebral arterial and venous flow. However, arteries and veins require different velocity encoding (VENC) settings for optimal velocity-to-noise ratio (VNR). Consequently, venous measurements using arterial-optimized VENC settings are subject to reduced VNR and require further evaluation. This study compared cerebral venous flow and pulsatility assessments using a high-VENC (110 cm/s, adapted to the arterial system) and a low-VENC (40 cm/s, adapted to the venous system) 4D-flow MRI sequence at 3 Tesla. Flow and pulsatility index (PI) were calculated for cerebral veins, sinuses and internal jugular veins in 36 elderly volunteers (79 ± 5 years). The high-VENC acquisitions allowed visualization of nearly all venous structures. Mean flow differences were small and the correlation, strong, when comparing both acquisitions across sinuses (*R* = 0.90–0.99, difference = -8–7%) and cortical veins (*R* = 0.93, difference = − 6%). Inflow-outflow differences at the confluence of sinuses were similar between acquisitions. PI showed moderate to strong agreement except in the straight sinus. Both the vein of Galen and the jugular veins suffered from aliasing in the venous VENC acquisitions. In summary, this study demonstrated that a VENC setting adapted for the arterial cerebral circulation was feasible for studying cerebral venous flow and pulsatility.

**Supplementary Information:**

The online version contains supplementary material available at 10.1038/s41598-025-28405-8.

## Introduction

Four-dimensional phase-contrast MRI (4D-flow) is an established and widely accepted method for evaluating cerebral blood flow. Although research has predominantly focused on the major cerebral arteries^1,2^, there is a growing interest in using 4D-flow to study the venous system^3,4^. Methods optimized for venous flow assessment have demonstrated high repeatability in cerebral sinuses^5^. 4D-flow MRI of arteries and veins requires the setting of velocity-encoding (VENC)—the user-defined parameter that sets the maximum measurable velocity without phase wrapping (aliasing). To optimize this setting, arterial and venous data are typically acquired in separate scans with different VENC values. However, if 4D-flow proves capable of accurately assessing both arterial and venous blood flow in a single acquisition, it would offer clear advantages.

Recent studies have examined the relationship between hemodynamic disturbances in the cerebral sinuses and neurological disorders, such as Alzheimer’s disease^6,7^, multiple sclerosis^8,9^, pulsatile tinnitus^10–12^, and idiopathic intracranial hypertension^13,14^. However, compared to arteries, venous flow acquisitions are typically expected to require a lower VENC to maintain a sufficient velocity-to-noise ratio (VNR). While arterial flow is typically assessed with VENCs up to 110 cm/s ^15–19^ studies focusing on the cerebral venous system use VENCs of 40 cm/s or lower^8,9,14,19–21^. The low VENC can become problematic when a comprehensive assessment of cerebral venous disorders is needed, particularly in cases involving both slow and fast-flowing segments, such as venous stenotic regions^10,13,22^, or for utilization of the simultaneous measurement of arterial and venous flow for assessment of inflow-outflow dynamics^19,21^, arterial-venous transit time^7,23^, and pulsatility^3,7^.

If the VENC is set too low, substantial aliasing can occur in the fast-flowing segments of the cerebral vasculature. However, while increasing the VENC can minimize aliasing in these high-velocity regions, it will reduce the VNR in slow-flowing regions of the vasculature, such as the venous system. Dual-VENC approaches have emerged lately to address this problem, but they typically result in longer scan times^24,25^.

A single scan using VENC parameters optimized for arterial flow velocities offers a time-efficient alternative. Although the resulting VNR is reduced due to the suboptimal VENC for venous flow, this limitation may be mitigated by averaging measurements across multiple consecutive cross sections along a vessel segment. However, the feasibility of this approach requires evaluation. Such evaluation is important in the design of new studies of cerebral hemodynamics but also for enabling analysis of venous hemodynamics, such as mean flow and pulsatility, in already available cohorts where cerebral arterial blood flow has been investigated.

This study aimed to evaluate the feasibility of analyzing cerebral venous flow parameters using acquisitions originally optimized for the arterial system. Specifically, we compared cerebral venous blood flow and pulsatility metrics obtained from two 4D-flow MRI sequences: a high-VENC setting of 110 cm/s (adapted to the cerebral arterial system) and a low-VENC setting of 40 cm/s (adapted to the cerebral venous system).

## Materials and methods

### Study participants

Thirty-seven elderly volunteers underwent an MRI scan protocol including two 4D-flow acquisitions with 110 cm/s VENC (VENC110) and 40 cm/s VENC (VENC40) respectively. One participant was excluded due to technical reasons. Risk factors included hypertension (*n* = 24), hyperlipidemia (*n* = 14), current smoking (*n* = 1), and former smoking (*n* = 6). Comorbidities included atrial fibrillation (*n* = 3), diabetes mellitus (*n* = 2), ischemic heart disease (*n* = 2), and cerebrovascular disease (*n* = 2). Two participants had Alzheimer’s disease. The final study population consisted of 36 participants (20 women and 16 men, aged 79 ± 5 years, ranging from 70 to 91 years).

### Ethical considerations

The study was approved by the Regional Ethical Review Board in Umeå, Sweden (Ref no. 2017/253 − 31), and conducted in accordance with the Declaration of Helsinki. All participants received both oral and written information about the study and provided informed consent prior to participation.

### MRI protocol

Both acquisitions were acquired consecutively in one session—VENC110 before VENC40—on a 3 Tesla GE Discovery MR750 (Milwaukee, WI/USA) scanner, using a 32-channel head coil. The 4D-flow data were acquired by a phase contrast vastly under sampled isotropic projection reconstruction (PCVIPR) sequence^26^ using the following parameters: TR/TE, 6.5/2.7 ms and 6.9/3.1 ms for VENC110 and VENC40, respectively; radial projections, 16,000; VENC, 40 cm/s and 110 cm/s (5-point); flip angle, 8°; matrix size, 320 × 320 × 320; isotropic resolution 0.7 × 0.7 × 0.7 mm³; temporal resolution, 20 cardiac phases. The acquisitions were second-order polynomial eddy current corrected during reconstruction. Reconstructed data comprised time-resolved 3D velocity volumes (no phase aliasing correction applied), complex-difference images, and T1-weighted magnitude images. Scan time was approximately 9 min per VENC acquisition.

### Measurement locations

**Fig. 1:**
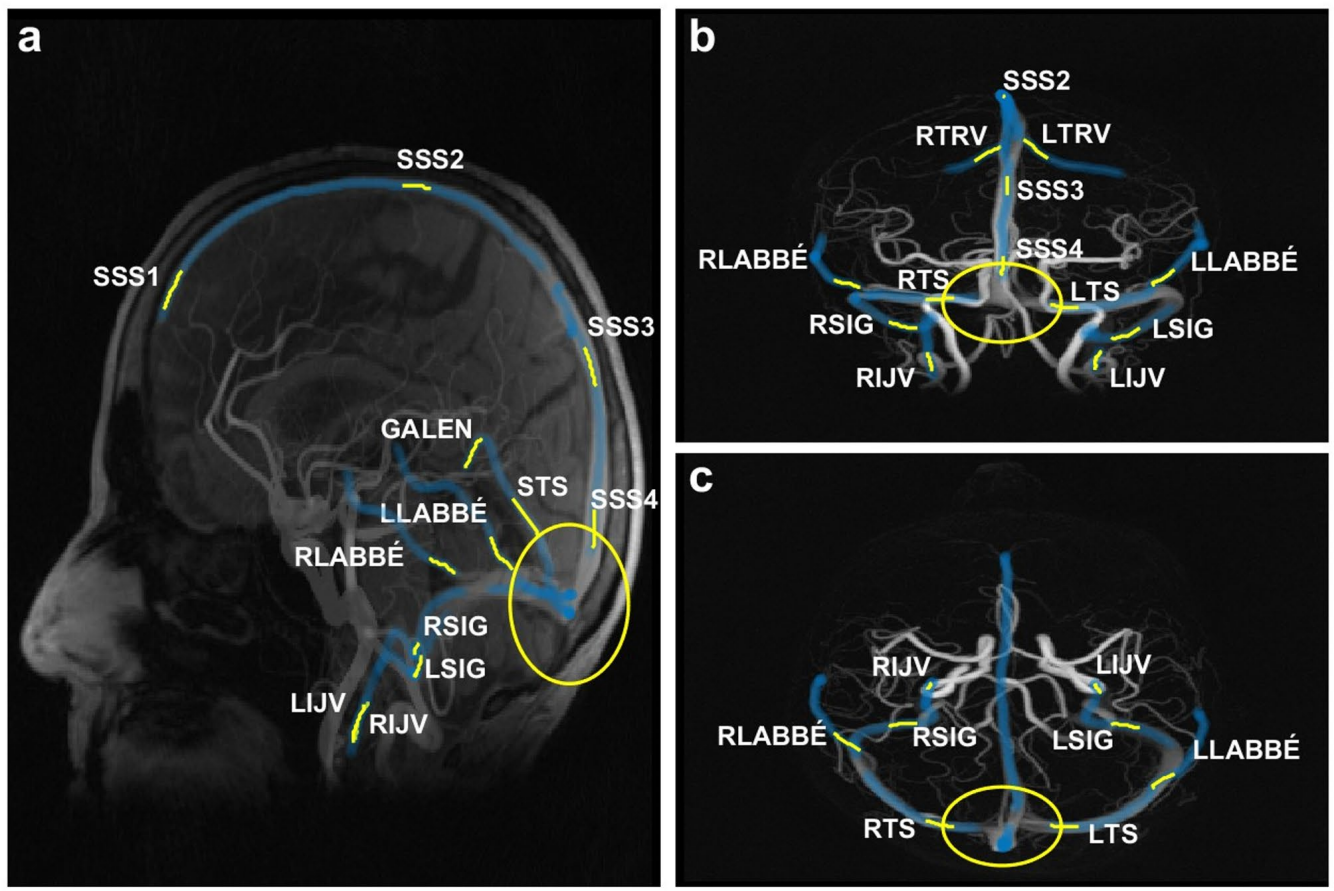
Maximum intensity projections from a 4D-flow acquisition (VENC110) illustrating typical measurement locations. (**a**) The sagittal projection (**b**) coronal projection and (**c**) the axial projection. Veins are illustrated in blue. Yellow lines indicate measurement locations and their associated cross section segments, and the yellow circle marks the measurement locations of SSS4, RTS and LTS. SSS1: anterior superior sagittal sinus, SSS2: mid superior sagittal sinus, SSS3: posterior superior sagittal sinus, SSS4: distal superior sagittal sinus, STS: straight sinus, RTS: right transverse sinus, LTS: left transverse sinus, RSIG: right sigmoid sinus, LSIG: left sigmoid sinus, RIJV: right internal jugular vein, LIJV: left internal jugular vein, GALEN: vein of Galen, TRV: vein of Trolard, LABBÉ: vein of Labbé.

Measurement locations were identified on the VENC110 complex-difference maximum intensity projection (MIP) angiograms overlaid with vessel centerlines. The centerlines of the vascular tree were extracted from the complex-difference volume using a thinning algorithm, leaving a 1-voxel-thick centerline tree^27,28^ representing the center of the cerebral vessels. To reduce noise, each measurement included a 15-voxel-long centerline segment^29^, symmetrically distributed over the location, from which the mean flow of the segment was used for the subsequent analysis. If the measurement location was placed near a centerline junction, the centerline segment comprised the 15 cross sections either proximal or distal to the junction, depending on which side of the junction the measurement location was placed. If the location was between two junction points that were less than 15 centerline points apart, the final segment contained fewer cross sections. The same locations were identified in the VENC40 acquisitions. The measurement locations at which mean flow and pulsatility index (PI) were assessed and compared between the two acquisitions are listed below, and shown in Fig. 1. To determine the number of venous structures missed by VENC110 but visualized with VENC40, we also inspected the VENC40 angiograms.

### Intracranial sinuses

The superior sagittal sinus (SSS) was assessed in 4 segments (SSS1-SSS4) which were defined as follows: the most rostral segment (SSS1), mid segment (SSS2) where the vertical line drawn from the posterior border of the brainstem intersected the SSS, and SSS3 at the occipital gyri in the midsagittal plane. SSS4, right transverse sinus (RTS), left transverse sinus (LTS) and the occipital sinus (OS) were assessed 2 cm from the point at the coronal and axial position of the confluence of sinuses (CoS) in the mid sagittal plane (Fig. 1a-c). The straight sinus (STS) and the sigmoid sinuses (RSIG, LSIG) were assessed at the mid segment.

### Internal jugular veins

The internal jugular veins (IJVs) were assessed as distally as possible on their associated centerline (Fig. 1a-c).

### Vein of Galen

The measurement location for the vein of Galen was defined as the approximately 1-centimetre-long, often upward-facing segment, proximal to origin of the STS (Fig. 1a). In cross sections showing multiple adjacent vessels with co-directional flow, most likely basal veins and internal cerebral veins, flow rates were summed to obtain total flow. The centerline section had to be shortened – in a few subjects down to 6 voxel points - in order not to include the proximal segment of the STS.

### Cortical veins

Cortical veins were identified in proximity to the SSS (typically the vein of Trolard labeled TRV, in Fig. 1b) and the LTS and RTS (typically the vein of Labbé labeled LABBÉ, in Fig. 1a-c) where any visible vessel centerline (in VENC110) that fed into these sinuses were investigated.

### Conservation of flow at the confluence of sinuses

To further evaluate the VENC110 acquisitions, conservation of flow in the CoS was assessed. Inflow and outflow were assessed and compared. Inflow was defined as the sum of flow of the inlets (typically SSS4 + STS). Outflow was defined as the sum of flow of the outlets (typically RTS, LTS and OS). If the sinuses were bifurcated, the flow rates in each branch were added to obtain the total flow of the vessel.

### Flow assessments

Flow assessments were conducted in MATLAB (The MathWorks Inc., R2022b, Natick, Massachusetts; https://www.mathworks.com). For each voxel along the centerline segment of the measurement location, a 24 × 24-voxel cross section was extracted perpendicular to the centerline direction. The vessel area was then initially segmented automatically and subsequently refined manually upon visual inspection to ensure accurate delineation of the vessel lumen. Cross sections with ambiguous boundaries were excluded from the centerline segment and omitted from further analysis. Assessments in which the centerline segment contained fewer than 5 cross sections were excluded from analysis. All measurements were performed by a single evaluator (A. Westlund).

The automatic segmentation approach was based on a previously validated method for cerebral arteries, where vessel areas were extracted using a local threshold of 16% of the maximum complex-difference value at the vessel center^29,30^. To reduce the need for manual corrections—particularly in vessels with lower flow velocities—this approach was extended by incorporating an additional threshold of 14% based on the peak time-averaged velocity of the cross section (see Supplementary Fig.S1 online). The combination of the two thresholds introduced a small average reduction of − 2.6 ± 1.9 mL/min compared to the original segmentation approach, while it reduced the number of manual corrections needed. Of the 6,843 cross sections from the VENC110 acquisitions, 69% required manual corrections using the original segmentation approach, whereas the proposed combined approach required manual corrections in 43.9% (see Supplementary Table S1 online). In contrast, the proportion of cross sections that required manual corrections in the VENC40 acquisitions was 20.9% using the proposed combined approach. The higher success rate of VENC40 is potentially explained by its superior VNR relative to VENC110. Because velocity scales with phase as $\:v=(\text{VENC}/\pi\:)\varphi\:$, the same phase noise $\:{\sigma\:}_{\varphi\:}$ produces smaller velocity fluctuations at VENC40 than at VENC110. Consequently, in VENC110 identical phase deviations map to larger apparent velocities, causing more voxels to exceed the automatic segmentation threshold.

The velocity vectors were multiplied by the voxel area and summed over the segmented vessel area. Mean flow was determined as the average flow over the cardiac cycle. Finally, flow was estimated by averaging across all cross sections that made up the centerline section of the measurement location.

To estimate PI the cardiac waveform from each cross section was filtered with MATLAB lowpass filter function using parameters validated for 4D-flow PI analysis in cerebral arteries^29^ (cut of frequency, 1.9 Hz and steepness 0.85) (se Supplementary Fig.S2 online). PI was then derived from the average filtered waveform of the centerline segment and was defined as

1 $$\:PI=\frac{Qmax-Qmin}{Qmean}$$

where $\:Qmax$, $\:Qmin$ and $\:Qmean$ represent maximum, minimum and mean flow respectively, during the cardiac cycle. Assuming parabolic flow, the maximum velocity for each cross section was estimated by multiplying the peak flow by 2 and dividing by the vessel cross-sectional area.

### Statistics

Mean flow rates and PI were compared between the two acquisitions using paired t-tests and evaluated for correlation with the Pearson correlation coefficient. Flow rate differences and correlations were visualized with Bland–Altman and correlation plots. Variables that failed the Lilliefors test for normality were reanalyzed with nonparametric alternatives: Wilcoxon signed-rank test for paired comparisons and Spearman’s ρ for associations. A sensitivity analysis was conducted comparing the number of cross sections for the VENC110 acquisitions using paired t-tests. A significance level of α = 0.05 was applied to all statistical analyses (Bonferroni-adjusted α = 0.0038 for multiple comparisons).

## Results

### Feasibility and correlation analysis

Flow assessments with VENC110 were feasible at 309 out of 318 (97%) measurement locations within the intracranial sinuses. Nine locations were not visualized with VENC110 but were assessable with VENC40 (1 SSS1, 4 LSIG, 4 LTS), for which average flow was 16.3 ± 6.4 mL/min. Two OS were visualized in both acquisitions. In 1 subject, the vein of Galen was not visualized in either acquisition. Cortical veins were more frequently visible in the VENC40 acquisitions (see Supplementary Fig.S3 online), but at least one cortical vein was assessable with VENC110 in all but one subject. The heart rate averaged 66 bpm during the VENC110 acquisition and 65 bpm during the VENC40 acquisition. On average, heart rate was 1 ± 2 bpm higher with VENC110 compared with VENC40 (*P* = 0.01).

**Fig. 2:**
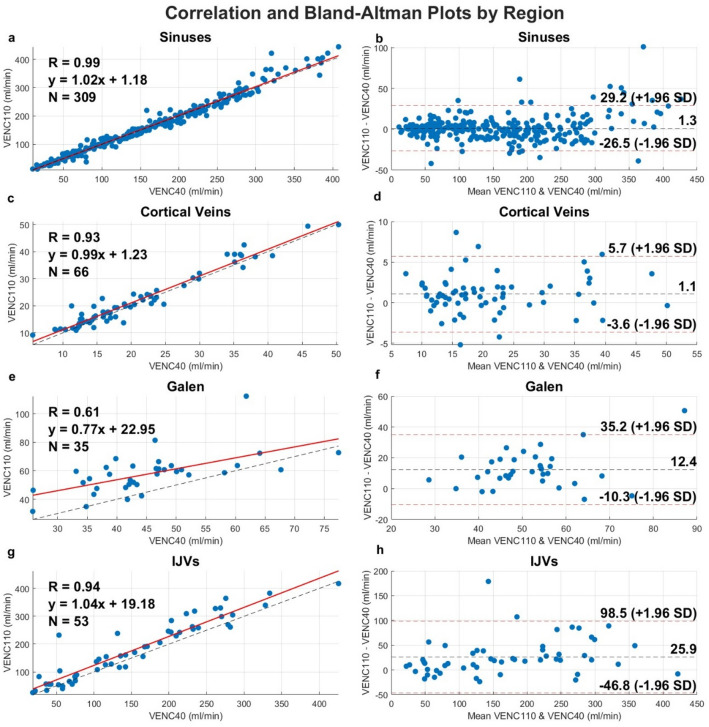
Correlation, R (Pearsons *r or* Spearman’s ρ depending on the distributional assumptions), and Bland Altman plots for separate venous vascular regions: Intracranial sinuses (**a**, **b**), cortical veins (**c**, **d**), vein of Galen (**e**, **f**) and IJVs (**g**, **h**).

Fig. 2 shows the correlation and Bland-Altman analyses for the intracranial sinuses, cortical veins, IJVs and the vein of Galen. In summary, the VENC40 and VENC110 acquisitions showed strong correlations in the intracranial sinuses (R_flow_ = 0.9–0.99) and cortical veins (R_flow_ = 0.93), with only minor differences in mean flow rates (VENC110 - VENC40), ranging from − 8% to 7% in the sinuses and − 6% in the cortical veins. In contrast, larger flow differences were observed in vein of Galen (difference = 24%, R_flow_ = 0.61) and in the IJVs (11–20% difference, R_flow_ = 0.91-0.96). Both these measurement locations exhibited one case each of visible aliasing (i.e., the presence of opposite color-codes within the vessel lumen on the velocity images) in the VENC40 acquisitions (see Supplementary Fig.S4 online).

The cross-sectional area measurements also showed small mean differences and strong correlations (R_area_ = 0.84–0.99) for all measurement locations except the cortical veins (R_area_ = 0.67).

The IJVs exhibited signal loss near the inferior boundary of the scan volume in several subjects and in both VENC acquisitions. To mitigate the impact of signal loss, only the 5 most cranial cross sections of the IJVs were included in the analysis. One LIJV was excluded due to having fewer than 5 reliable cross sections.

Table 1 summarizes the quantitative results from all vessel segments assessed with the VENC110 acquisition, including mean flow rates, relative differences between VENC40 and VENC110 (VENC110 – VENC40), correlation coefficients, mean cross-sectional areas and estimated maximum velocities.

**Table 1:** Flow (mL/min), area (mm²), maximum velocity (cm/s), and absolute/relative differences between VENC110 and VENC40, with paired-comparison p-values (P_flow_, P_area_) and correlation coefficients (R_flow_, R_area_) for all measurement locations identified from the VENC110 acquisitions.

Vessel	Flow110	Flow40	FlowDiff	FlowDiff %	*P*_flow_	*R*_flow_	Area110	Area40	AreaDiff	*P*_area_	*R*_area_	MaxVel110	MaxVel40	*N*
SSS1	33.8 ± 8.1	32.6 ± 8.9	1.2 ± 3.8	4.5 ± 11.1	0.08	0.9	11.2 ± 1.9	11.6 ± 2.5	− 0.4 ± 1.4	0.39^np^	0.84^np^	13.5 ± 3.6	12.9 ± 3.8	35
SSS2	95.7 ± 21.7	93.5 ± 21.4	2.2 ± 6.5	2.4 ± 6.7	0.05	0.95	17.5 ± 3.2	18.7 ± 4.0	− 1.3 ± 1.5	< 0.01	0.93	24.7 ± 5.2	22.0 ± 4.8	36
SSS3	194.4 ± 43.3	202.8 ± 48.5	− 8.4 ± 9.7	− 4.0 ± 4.1	< 0.01^np^	0.95^np^	26.9 ± 3.6	27.9 ± 4.1	− 1.0 ± 1.2	< 0.01^np^	0.96^np^	31.3 ± 6.4	30.8 ± 5.5	36
SSS4	216.9 ± 48.0	218.4 ± 48.8	− 1.5 ± 10.1	− 0.5 ± 4.5	0.38	0.98	33.0 ± 6.8	34.6 ± 7.4	− 1.6 ± 2.1	< 0.01^np^	0.96^np^	29.2 ± 6.0	27.6 ± 5.5	36
STS	64.8 ± 13.6	60.5 ± 13.3	4.3 ± 5.2	7.0 ± 8.6	< 0.01	0.92	12.7 ± 2.0	13.0 ± 2.5	− 0.2 ± 1.0	0.36^np^	0.94^np^	22.6 ± 5.6	19.4 ± 3.5	36
RTS	231.9 ± 98.6	224.3 ± 94.4	7.5 ± 14.2	3.5 ± 7.3	< 0.01	0.99	42.1 ± 13.8	42.0 ± 13.2	0.1 ± 3.6	0.79^np^	0.97^np^	24.2 ± 7.9	23.0 ± 7.2	35
LTS	115.9 ± 75.9	121.2 ± 76.1	− 5.3 ± 11.8	− 6.2 ± 20.7	0.03^b, np^	0.98^np^	29.4 ± 12.2	31.2 ± 12.1	− 1.8 ± 2.9	< 0.01	0.97	16.0 ± 6.3	15.8 ± 5.6	26
OS	64.5 ± 43.8	53.5 ± 26.7	11.1 ± 17.2	12.2 ± 21.8	N/A	N/A	12.7 ± 2.1	13.0 ± 2.1	− 0.3 ± 0.0	N/A	N/A	22.4 ± 12.2	17.7 ± 4.4	2
RSIG	241.1 ± 101.5	225.8 ± 90.6	15.3 ± 25.4	6.0 ± 10.6	< 0.01^np^	0.98^np^	37.1 ± 9.7	39.1 ± 9.8	− 2.0 ± 2.7	< 0.01	0.96	27.3 ± 7.6	23.7 ± 5.9	36
LSIG	126.9 ± 78.8	133.7 ± 77.6	− 6.8 ± 10.5	− 8.4 ± 12.0	< 0.01	0.99	26.7 ± 9.5	28.9 ± 9.7	− 2.2 ± 2.4	< 0.01	0.97	18.4 ± 6.4	17.9 ± 5.7	31
RIJV*	230.0 ± 102.5	191.7 ± 96.9	38.4 ± 41.9	20.3 ± 25.1	< 0.01	0.91	49.4 ± 24.3	49.4 ± 23.2	− 0.1 ± 4.8	0.95	0.98	22.4 ± 5.7	17.0 ± 4.4	30
LIJV*	118.8 ± 76.8	109.2 ± 74.5	9.6 ± 21.1	11.0 ± 29.7	0.04^b^	0.96	32.5 ± 21.3	34.9 ± 19.6	− 2.5 ± 4.8	0.01^b, np^	0.99^np^	18.0 ± 6.2	13.6 ± 3.6	23
GALEN	57.9 ± 14.2	45.5 ± 11.2	12.4 ± 11.6	23.9 ± 19.4	< 0.01	0.61	13.6 ± 2.7	14.9 ± 3.5	− 1.3 ± 2.0	< 0.01	0.82	18.4 ± 4.7	12.6 ± 2.4	35
CORTICAL VEINS	21.7 ± 10.0	20.6 ± 9.8	1.1 ± 2.4	5.9 ± 13.9	< 0.01^np^	0.93^np^	9.7 ± 1.7	10.0 ± 2.1	− 0.3 ± 1.6	0.11	0.67	9.5 ± 3.1	8.6 ± 2.9	66

P-values are from paired t-tests or Wilcoxon signed-rank tests as appropriate; correlations are Pearson’s *r* or Spearman’s ρ depending on distributional assumptions.

SSS1: anterior superior sagittal sinus, SSS2: mid superior sagittal sinus, SSS3: posterior superior sagittal sinus, SSS4: distal superior sagittal sinus, STS: straight sinus, RTS: right transverse sinus, LTS: left transverse sinus, RSIG: right sigmoid sinus, LSIG: left sigmoid sinus, RIJV: right internal jugular vein, LIJV: left internal jugular vein, GALEN: vein of Galen, CORTICALS VEINS: Trolard and Labbé veins.

*Analysis made on the 5 most cranial cross section.

^b^Non-significant with Bonferroni adjusted p-value.

^np^Nonparametric test value (Signed-rank P-value or Spearman’s ρ).

### Conservation of flow in confluence of sinuses (CoS)

**Fig. 3:**
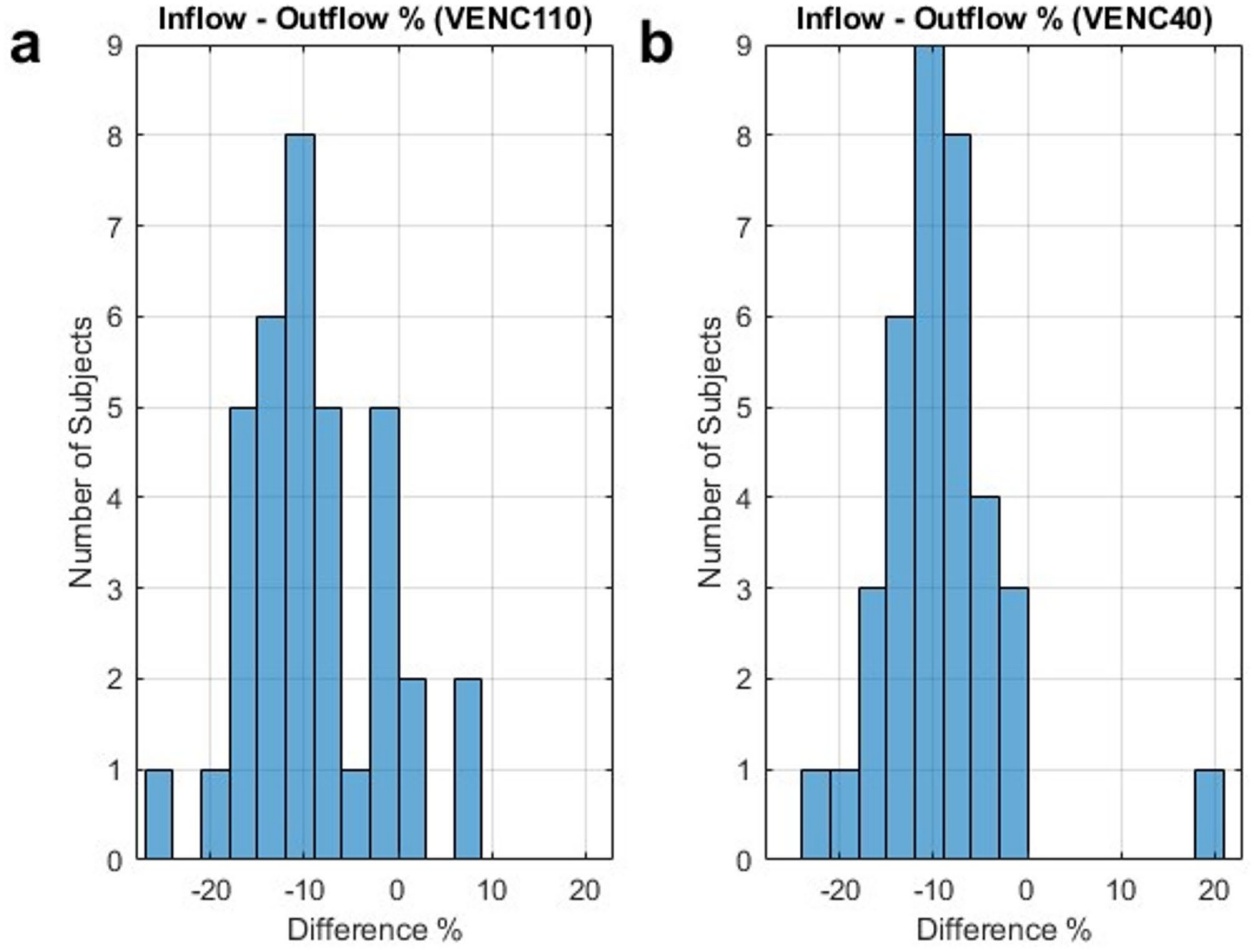
Relative difference between inflow and outflow in the confluence of sinuses for VENC110 (**a**) and VENC40 (**b**).

The net difference between total cerebral venous inflow and outflow was comparable between the two acquisitions (*p* = 0.79), with a mean imbalance of − 8.8 ± 7.1% for VENC110 and − 9.0 ± 6.8% for VENC40 (Fig. 3). When the STS was instead assessed as close as possible to the CoS, this flow mismatch was reduced, yielding a mean difference of -4.3 ± 5.9% for VENC110 and − 3.8 ± 6.5% for VENC40. Additionally, 4 LTS demonstrated reversed flow into the CoS. Of these, 2 were only visible with VENC40. Both OS drained in the direction toward the marginal sinuses and the jugular bulb.

### Pulsatility index

The PI did not differ between the VENC40 and VENC110 acquisitions for any vessel except in the STS, where a significant difference was observed, *p* = 0.01 (non-significant after Bonferroni adjusted p-value). Correlation coefficient (R) for PI ranged from 0.23 to 0.86 across the intracranial sinuses, 0.54 in the cortical veins, 0.50–0.72 in the IJVs, and 0.04 in the vein of Galen. Detailed results are presented in Table 2.

**Table 2:** Pulsatility index (PI) and PI-differences between VENC110 and VENC40, with paired-comparison p-values (P) and correlation coefficients (R) for all measurement locations identified in the VENC110 acquisitions.

Vessel	PI-110	PI-40	PI-DIFF	*P*	*R*	*N*
SSS1	0.60 ± 0.23	0.63 ± 0.27	-0.03 ± 0.21	0.36	0.66	35
SSS2	0.65 ± 0.24	0.60 ± 0.26	0.05 ± 0.21	0.14	0.65	36
SSS3	0.59 ± 0.24	0.55 ± 0.20	0.04 ± 0.15	0.16	0.78	36
SSS4	0.61 ± 0.28	0.57 ± 0.23	0.03 ± 0.16	0.2	0.83	36
STS	0.57 ± 0.22	0.45 ± 0.19	0.12 ± 0.26	0.01^b^	0.23	36
RTS	0.59 ± 0.24	0.58 ± 0.19	0.01 ± 0.15	0.67	0.77	35
LTS	0.69 ± 0.64	0.76 ± 0.90	-0.07 ± 0.34	0.77^np^	0.86^np^	26
OS	0.87 ± 0.20	0.78 ± 0.33	0.10 ± 0.13	N/A	N/A	2
RSIG	0.57 ± 0.19	0.55 ± 0.19	0.02 ± 0.14	0.31	0.73	36
LSIG	0.53 ± 0.23	0.49 ± 0.22	0.04 ± 0.22	0.31	0.54	31
RIJV*	0.78 ± 0.36	0.86 ± 0.46	-0.08 ± 0.42	0.39^np^	0.72^np^	30
LIJV*	0.82 ± 0.39	0.71 ± 0.35	0.11 ± 0.37	0.17	0.5	23
GALEN	0.44 ± 0.17	0.43 ± 0.25	0.01 ± 0.29	0.27^np^	0.04^np^	35
CORTICAL VEINS	0.57 ± 0.27	0.55 ± 0.29	0.02 ± 0.27	0.49	0.54	66

P-values are from paired t-tests or Wilcoxon signed-rank tests, as appropriate; correlations are Pearson’s *r* or Spearman’s ρ depending on distributional assumptions.

SSS1: anterior superior sagittal sinus, SSS2: mid superior sagittal sinus, SSS3: posterior superior sagittal sinus, SSS4: distal superior sagittal sinus, STS: straight sinus, RTS: right transverse sinus, LTS: left transverse sinus, RSIG: right sigmoid sinus, LSIG: left sigmoid sinus, RIJV: right internal jugular vein, LIJV: left internal jugular vein, GALEN: vein of Galen, CORTICAL VEINS: Trolard and Labbé veins.

*Analysis made on the 5 most cranial cross sections.

^b^Non-significant with Bonferroni adjusted p-value.

^np^Nonparametric test value (Signed-rank P-value or Spearman’s ρ).

### Cross section sensitivity analysis

There were no differences in flow rate using the 5 most centered cross sections or all available 15 cross sections in the intracranial sinuses (-0.1 ± 3.4 mL/min, *p* = 0.5), cortical veins (-0.1 ± 1.5 mL/min, *p* = 0.6). For the vein of Galen, too few subjects had 15 cross sections available. Consequently, the comparison was made between 10 and 5 cross sections to ensure adequate statistical power (–0.4 ± 4.5 mL/min, *p* = 0.7). These results support the inclusion of vessels with as few as 5 cross sections in the main analysis.

## Discussion

This study demonstrated that 4D-flow MRI with a VENC optimized for cerebral arterial flow (110 cm/s) yielded flow measurements in the intracranial venous sinuses and cortical veins that were comparable to those obtained with a typical venous-optimized VENC (40 cm/s). The arterial VENC acquisition (VENC110) was sufficient for detection of nearly all measurement locations identified with the venous VENC setting (VENC40). For both the intracranial sinuses and cortical veins, the two acquisitions showed strong agreement with minimal differences. The conservation-of-flow analysis demonstrated relatively strong agreement between inflow and outflow at the confluence of sinuses, with a bias toward greater outflow. Including the distal segment of the STS in the analysis reduced the inflow–outflow imbalance, suggesting that the observed outflow bias likely reflects additional venous tributaries entering the sinuses between STS/SSS4 and RTS, LTS and OS measurement locations.

These findings address a knowledge gap regarding the suitability of arterial VENC settings for analyzing cerebral venous hemodynamics. The results suggest that a single high-VENC acquisition can be used to adequately capture venous dynamics, relaxing the need for separate acquisitions for arterial and venous analysis. Importantly, this approach simplifies exploration of venous flow disturbances, both in new studies and in retrospective venous analyses of previously acquired datasets collected with arterial settings. This is particularly significant for studies requiring a comprehensive and simultaneous assessment of cerebral hemodynamics, such as studying arterial-venous transit time or the distribution of venous outflow relative to arterial cerebral inflow.

A novelty in this work included the presentation of typical flow rates for the larger cortical veins, not previously reported. This expands the applicability of 4D-flow MRI to vessels in which flow quantification has traditionally been limited due to anticipated technical challenges, such as low flow velocities. The ability to capture reliable flow data in these smaller veins, which are surrounded by cerebrospinal fluid (CSF), opens new opportunities for investigating their roles in both cerebral hemodynamics and as a source of compliance in CSF dynamics.

The mean flow differences between the two acquisitions fell within the range typically expected for within-subject physiological variation in cerebral blood flow^31,32^ and in SSS flow^5^. The flow rates in the intracranial sinuses aligned with previous 4D-flow assessments^3,4,7^, with minor discrepancies likely due to age differences and slight variations in measurement locations. However, the combined flow rates of SSS4 and STS were lower than those reported by Lokossou et al. ^33^, which used two-dimensional phase contrast (2DPCMRI) and included a slightly younger cohort. In addition, the measurement planes in the previous study were placed in non-perpendicular orientations relative to STS and SSS which has shown to overestimate blood flow^34^.

The IJVs and the vein of Galen each exhibited one case of suspected aliasing in the VENC40 acquisitions, suggesting that flow velocities in these vessels may approach the upper measurable limit of VENC40. This issue was not reflected in the estimated maximum velocities, likely because the assumption of a parabolic flow profile was not valid in these regions. In addition, the region of the vein of Galen was more challenging to clearly discern compared to the other investigated vessels. The vein is formed by the complex convergence of several veins^35^, most commonly the internal cerebral veins and basal veins, and terminates in the straight sinus where also the inferior sagittal sinus enters. This regional variability was evident as comparatively greater flow variation between cross sections observed in the vein of Galen cross-section sensitivity analysis. In addition, narrowing of the vein of Galen, as well as the presence of arachnoid granulations near the origin of the STS, are common^35,36^. When only including the five first cross-sectional planes in effort to avoid the narrow region, the R_flow_ increased from 0.61 to 0.84 which corresponded to an increase in mean flow in the VENC40 acquisitions, from 45.5 ± 11.2 mL/min to 53.2 ± 14.4 mL/min while VENC110 remained unchanged. These results suggest that only the very proximal portion of the vein should be assessed if using a low VENC (40 cm/s in this study) to avoid aliasing.

Assessment of the IJVs was affected by signal loss in both acquisitions which were partially recovered by restricting analysis to the five most cranial cross sections. Nevertheless, signal loss remained evident in several subjects, indicated by a marked decrease in flow along the centerline segment, from the most cranial to the most caudal cross sections. In addition, the jugular veins exhibited vortical flow patterns previously described^4,37,38^, that gave rise to misaligned centerlines for a number of subjects in both acquisitions. This made it difficult to distinctly delineate the IJVs in several cases, potentially due to misalignment between the centerline direction and the flow direction. Future work will include manual corrections of misaligned centerlines to improve the reliability of IJV assessments near the jugular bulb.

Nevertheless, the presence of aliasing in the vein of Galen and the IJVs highlights the wide range of flow velocities within the cerebral venous system, emphasizing the need for higher VENC settings, dual-VENC approaches, or anti-aliasing postprocessing. Here, we show that a single high-VENC acquisition is a simple, feasible option.

This study demonstrated moderate-to-high agreement in PI between VENC110 and VENC40 (except in the STS and vein of Galen), with correlations in the same range as previously reported between 4D-flow and 2DPCMRI for arteries^29^. The weak correlation in the vein of Galen is likely explained by aliasing, whereas the reason in the STS is unclear. The lack of differences in mean PI between VENC110 and VENC40 indicates that the two acquisitions overall yield similar PI estimates. However, within-subject variability in PI was large and approached between-subject variability. This pattern, as reflected in the weaker correlations, suggests reduced repeatability of venous PI estimates from 4D-flow. This limitation should be considered when designing studies investigating PI physiology.

The combined PI values for the SSS4 and STS agreed well with those reported in Lokossou et al. ^33^ for a slightly younger cohort. For 4D-flow, low-pass filtering is needed to reduce high-frequency noise at systolic peaks and diastolic troughs (both of which inflate PI) thereby mitigating falsely elevated PI estimates. The cutoff frequency of 1.9 Hz used for lowpass-filtering in this study has been shown to yield PI agreement between 4D-flow and 2DPCMRI in cerebral arteries^29^. The agreement of the PI findings supports the use of 1.9 Hz as cut of frequency for 4D-flow in the intracranial sinuses. Compared with previously reported 4D-flow data for the same age group, assessed without low-pass filtering of the waveforms, our PI values for the intracranial sinuses (SSS4,STS, RTS, LTS), were lower^3^ or similar^7^. Without filtering the waveform, our mean PI increased from 0.62 to 0.78 in the intracranial sinuses positioning our PI values between those reported in the two previous studies. Our PI values where higher than those presented in Dai et al. ^4^, although they used a lower temporal resolution and studied a younger cohort.

The cortical veins showed higher PI in this study than those reported by Driver et al. ^20^, who studied a younger cohort. Additionally, unlike the previous study, a higher VENC was used to avoid aliasing, and segmentation was performed on cross sections perpendicular to the vessel’s direction, enhancing reliability.

Overall, we show comparable PI values in the intracranial sinuses and the cortical veins when comparing an arterial - and a venous-optimized VENC. Our results, together with previously validated PI values for arteries^29^ mean that it is possible to study blood flow pulsatility as it propagates from the arterial inflow through the cortical veins and into the major venous outflow tracts of the intracranial sinuses. The blood flow pulsatility in cerebral veins is sourced by two components: the pulsatile arterial flow, which is dampened prior to the capillary outflow, and the intracranial pressure (ICP) variations caused by arterial expansion during each heartbeat, compressing elastic cerebral veins generating pulsatile venous flow^39^. Reliable methods for the simultaneous assessment of pulsatile arterial and venous flow help to explore the interplay of these mechanisms and their relevance to neurological diseases^7,23,40^.

This study has several limitations. First, vessels with low flow velocities show reduced visibility and may remain undetected in VENC110 acquisitions, a limitation particularly evident in cortical veins where many vessels could not be assessed. Consequently, the VENC110 analysis cannot be expected to have the same coverage as VENC40 when the objective is to study flow in cortical veins or other low-velocity venous structures. Second, the performance of the automatic segmentation method may vary in cohorts whose age distribution or health characteristics differ from those of the present study. Third, since every automatically segmented cross section was inspected (successfully segmented or manually refined) by the same operator, operator-related bias in the comparison between VENC110 and VENC40, should be limited. However, operator bias may be a concern if comparing flow assessments across studies with different operators. Fourth, phase aliasing correction was not applied during post-processing. Because aliasing was visually detected in the VENC40 acquisitions—for example, in the vein of Galen and the jugular veins—post-processing anti-aliasing^41^ might have reduced the observed flow-rate differences between VENC110 and VENC40.

In conclusion, this study demonstrated that arterial-optimized VENC settings (110 cm/s) can be reliably used for 4D-flow assessments of intracranial venous structures, including the dural sinuses and cortical veins. This allows simultaneous evaluation of cerebral arterial and venous flow from a single acquisition facilitating a more comprehensive analysis of cerebral hemodynamic disorders.

## Supplementary Information

Below is the link to the electronic supplementary material.

Supplementary Material 1

## Data Availability

Data from the corresponding author is available upon reasonable request.
