# Supplementary material for: Arterial-optimized 4D-flow MRI for quantifying flow and pulsatility in venous sinuses and large cerebral veins
Source: Sci Rep. 2025 Nov 20;15:41181. doi: 10.1038/s41598-025-28405-8 (PMC12639086; doi:10.1038/s41598-025-28405-8)
Supplement: Supplementary file 1 — Supplementary Material 1 [file 41598_2025_28405_MOESM1_ESM.pdf]

Supplementary

Automatic segmentation method

In addition to a complex difference threshold, voxels in the periphery of the vessel lumen were excluded based on a threshold determined relative to the maximum velocity at the vessel center, Thus, the segmentation assumes that velocities follow a parabolic flow profile and are aligned with the flow direction. This contrasts with segmentation based solely on complex difference images, which is insensitive to flow direction. This was empirically found to be beneficial in regions of slow flow and where adjacent vessels were present (Figure S1). However, by definition, the velocity-based thresholding method also includes only positive random noise, potentially leading to an overestimation of flow. To mitigate this, the velocity-based mask was combined with the original 16% complex difference threshold using the logical intersection of the two.

The velocity-based threshold was determined experimentally by comparing flow measurements - using different threshold - across cross sections where no manual adjustments had been required using the 16% complex difference approach (reference method). A threshold of 14% was found to minimize both the mean flow difference and standard deviation, resulting in a deviation of −0.7 ± 3 ml/min compared to the reference. The intersection of the two thresholds introduced a small average reduction of −2.6 ± 1.9 ml/min. Out of 6843 cross sections 69% needed manual corrections using the reference segmentation approach whereas the same number for the proposed combined approach was 43.9%. Table S1 show the results for each vessel specifically.

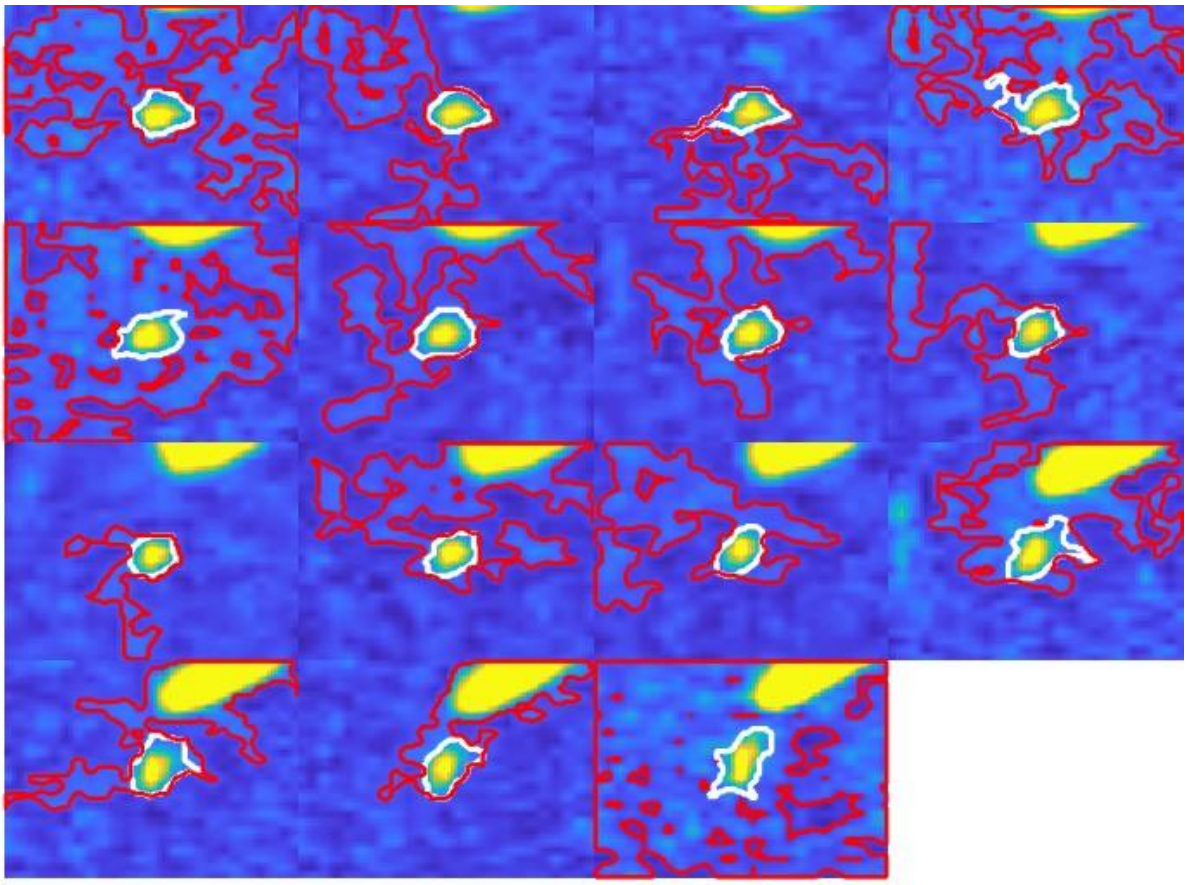

Fig.S1. Complex difference images of fifteen cross sections from a vein of Labbe' assessment showing segmentation results from the original approach (red), using 16% threshold on the complex difference maximum values, and from the combined approach (white) where a 14% threshold of the peak time-average velocity was added as well. Voxels with anti-parallel velocities in the surrounding, which includes the transverse sinus, are successfully eliminated.

Table S1. Percentages of automatically segmented cross sections in VENC110 acquisitions using the original segmentation approach (CD16 Auto) and the proposed segmentation approach (CD16 + Vel14 Auto); percentages of manually segmented and omitted cross sections with the proposed approach; and the mean number of cross sections per subject (nCross) used in the analysis.

Vessel	CD16 Auto %	CD16+Vel14 Auto %	Manual %	Omitted %	nCross	N
SSS1	21.5	58.9	38.7	2.5	14.6	35
SSS2	5.2	33	63.7	3.3	14.5	36
SSS3	86.1	94.3	5.7	0	15	36
SSS4	93.2	96.6	3.2	0.2	15	36
STS	29.4	73	26.9	0.2	15	36
RTS	71.5	86.2	13.8	0	14.9	35
LTS	27.9	62.3	37.7	0	15	26
OS	33.3	56.7	40	3.3	14.5	2
RSIG	43.4	54.5	45	0.6	14.9	35
LSIG	6.9	49.9	47.6	2.5	14.6	29
RIJV	1.1	20	79.1	0.9	14.7	30
LIJV	3.4	5.6	80.4	14	12.8	24
GALEN	3.9	34.8	57.8	7.4	11.6	35
CORTICALVEINS	2.8	42	53.6	4.3	14	66

SSS1: anterior superior sagittal sinus, SSS2: mid superior sagittal sinus, SSS3: posterior superior sagittal sinus, SSS4: distal superior sagittal sinus, STS: straight sinus, RTS: right transverse sinus, LTS: left transverse sinus, RSIG: right sigmoid sinus, LSIG: left sigmoid sinus, RIJV: right internal jugular vein, LIJV: left internal jugular vein, GALEN: vein of Galen, CORTICAL VEINS: Trolard and Labbe veins.

Lowpass filtering of waveforms

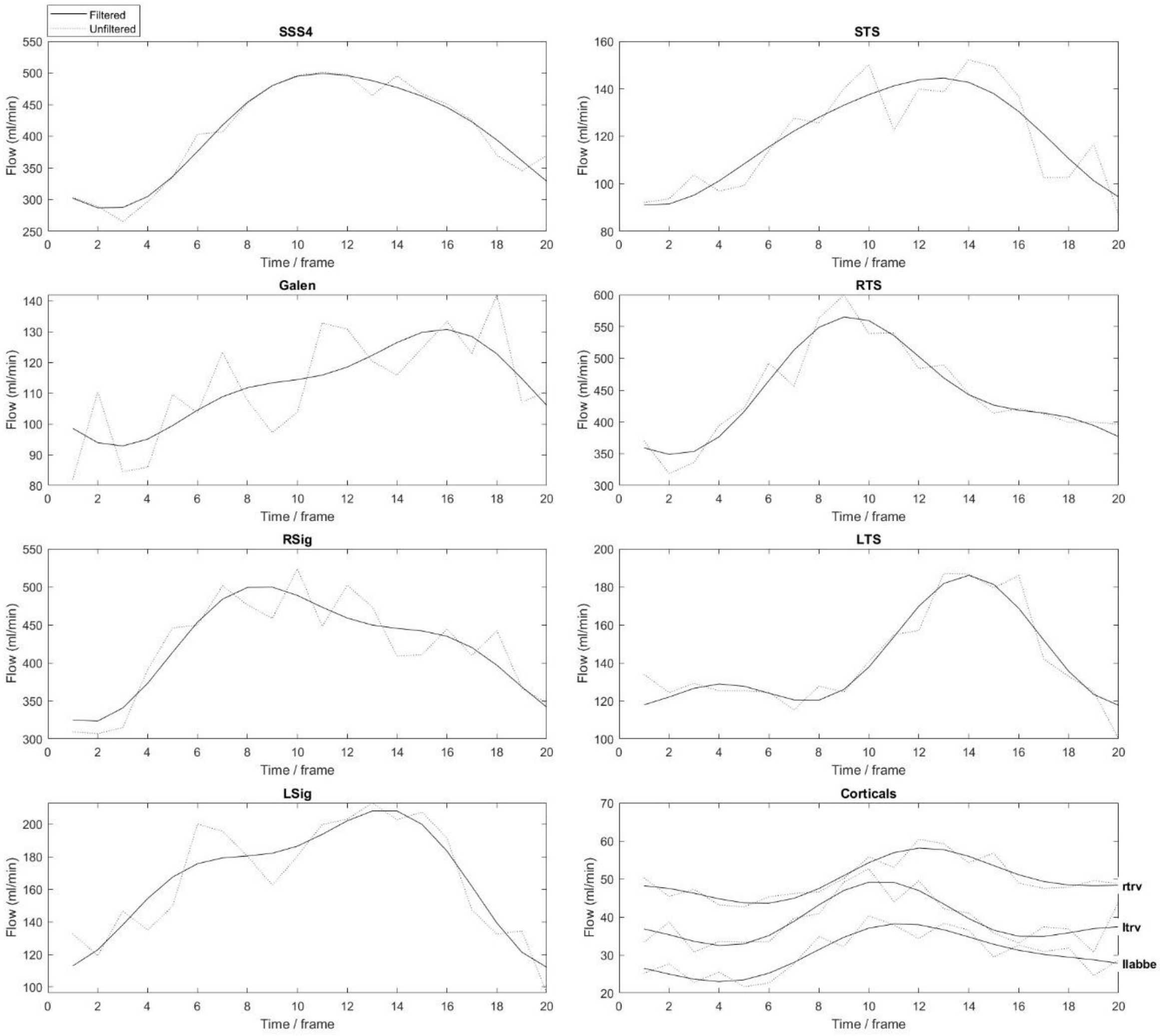

Fig.S2. The figure shows both the original waveform (dotted line) and the filtered waveform (solid line) at several measurement locations in one subject.

VENC110 and VENC40 complex-difference MIP

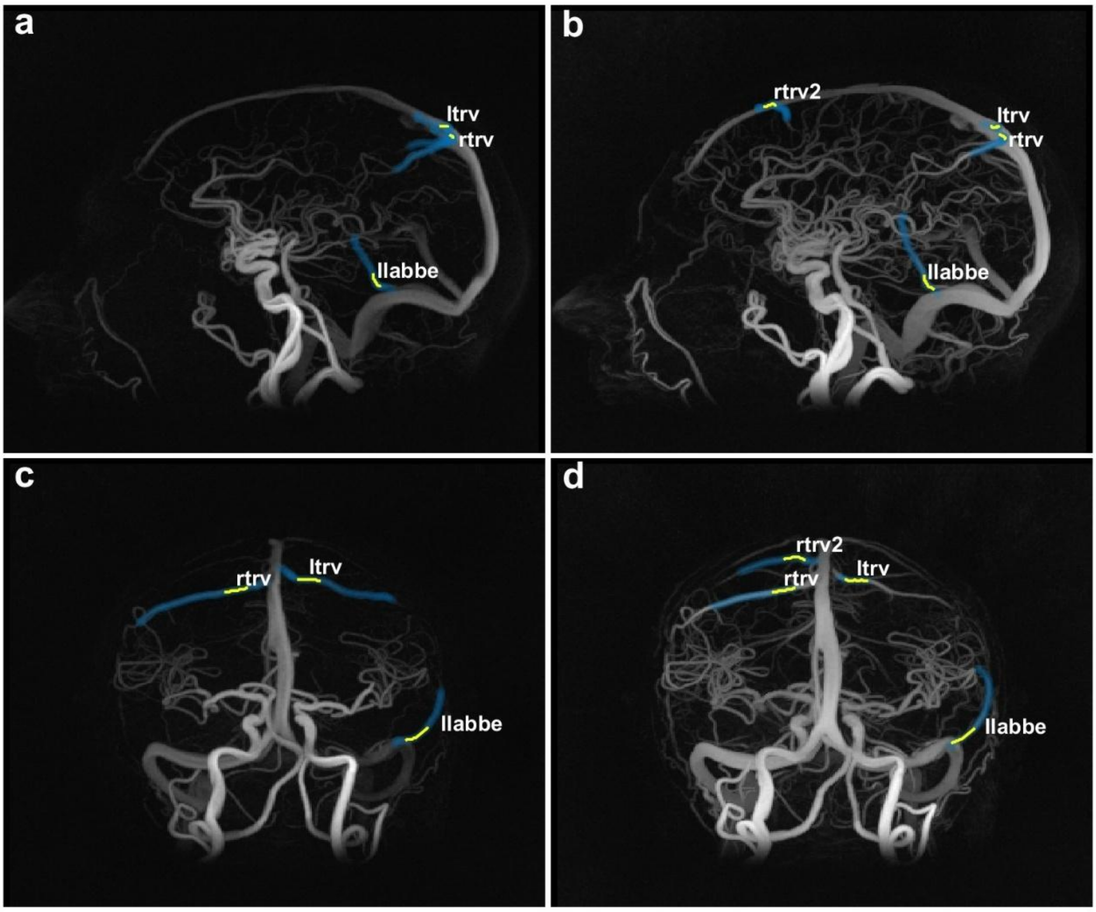

Fig.S3. Sagittal and coronal view of the maximum intensity projections of one subject: VENC110 (a,c) and VENC40 (b,d). The VENC40 acquisition were able to identify an additional cortical vein, “rtrv2”, that could not be identified in the VENC110 acquisition.

Aliasing in the vein of Galen and IJVs

Visible aliasing was identified in the vein of Galen and the IJVs in the VENC40 acquisitions in two subjects.

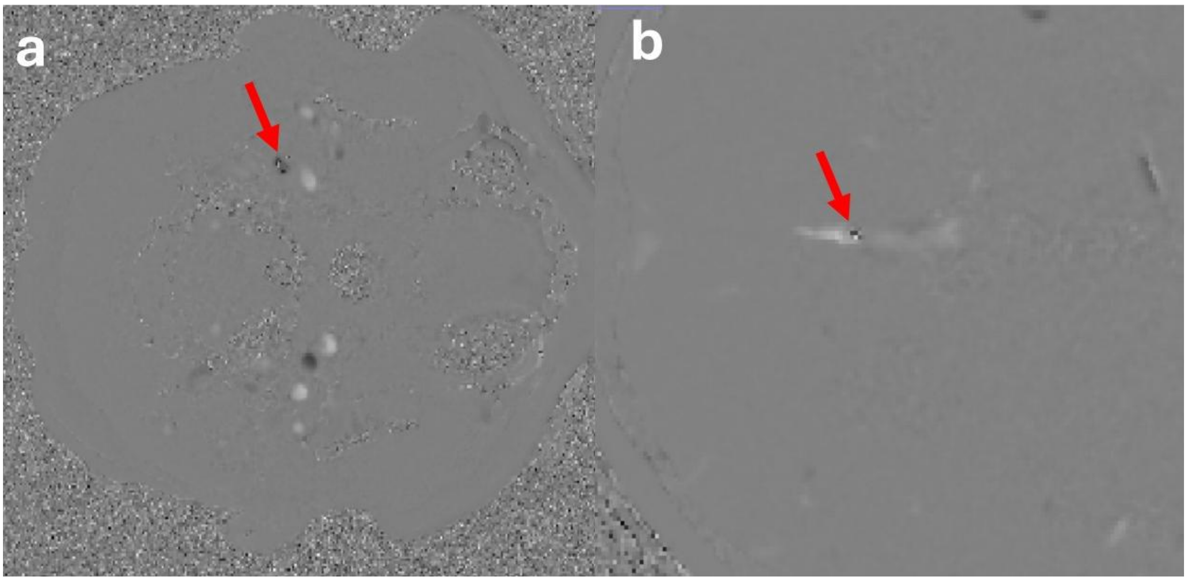

Fig.S4. Axial velocity images from the 40cm/s VENC acquisitions showing aliasing (red arrows) in a) the right internal jugular vein and b) the vein of Galen.
